# Anti-Diabetic Potential of Noni: The Yin and the Yang

**DOI:** 10.3390/molecules201017684

**Published:** 2015-09-25

**Authors:** Pratibha V. Nerurkar, Phoebe W. Hwang, Erik Saksa

**Affiliations:** Laboratory of Metabolic Disorders and Alternative Medicine, Department of Molecular Biosciences and Bioengineering, College of Tropical Agriculture and Human Resources, University of Hawaii at Manoa, Honolulu, HI 96822, USA; E-Mails: pwnhwang@hawaii.edu (P.W.H.); esaksa@hawaii.edu (E.S.)

**Keywords:** *Morinda citrifolia*, noni, Tahitian noni juice, type 2 diabetes, herbal products, alternative medicine

## Abstract

Escalating trends of chronic diseases such as type-2 diabetes (T2D) have sparked a renewed interest in complementary and alternative medicine, including herbal products. *Morinda citrifolia* (noni) has been used for centuries by Pacific Islanders to treat various ailments. Commercial noni fruit juice has been marketed as a dietary supplement since 1996. In 2003, the European Commission approved Tahitian noni juice as a novel food by the Health and Consumer Protection Directorate General. Among noni’s several health benefits, others and we have demonstrated the anti-diabetic effects of fermented noni fruit juice in animal models. Unfortunately, noni’s exciting journey from Polynesian medicine to the research bench does not reach its final destination of successful clinical outcomes when translated into commercial products. Noni products are perceived to be safe due to their “natural” origin. However, inadequate evidence regarding bioactive compounds, molecular targets, mechanism of action, pharmacokinetics, long-term safety, effective dosages, and/or unanticipated side effects are major roadblocks to successful translation “from bench side to bedside”. In this review we summarize the anti-diabetic potential of noni, differences between traditional and modern use of noni, along with beneficial clinical studies of noni products and challenges in clinical translation of noni’s health benefits.

## 1. Introduction

Incidence of chronic inflammatory metabolic disorders such as type-2 diabetes (T2D) is escalating worldwide. Prevalence of T2D is expected to increase from 2.8% in 2000 to 4.4% by 2030 [1]. Similarly, T2D-associated deaths are expected to double worldwide, from 2005 to 2030 [2]. In the United States, 25.8 million, or 8.3% of the population, including children and adolescents, suffer from T2D [3]. More often metabolic disorders including dyslipidemia, hypertension or vascular endothelial dysfunction also accompany T2D possibly leading to micro- and macro-vascular complications [4]. Although lifestyle changes such as diet and exercise are cornerstones of T2D treatment, the majority of individuals require pharmacological intervention that may include metformin, sulfonylureas, thiazolidinediones or glucagon-like peptide-1 agonists [5]. However, undesirable side effects of these anti-diabetic drugs, such as weight gain, hypoglycemia, and/or secondary failure possibly accelerated the resurgence of complementary and alternative medicine [6]. Natural products have emerged as one of the lucrative components of pharmaceutical industries generating more than $28 billion in revenues, globally.

Worldwide sales for one of the most popular natural product, *Morinda citrifolia L.* (Rubiaceace) commonly known as “noni”, is estimated at more than $2 billion dollars, which has increased from approximately $33 million to $400 million from 1999 to 2000 [7]. Noni is believed to have originated in Southeast Asia and subsequently distributed to the Western Pacific. Noni did not significantly influence ancient Polynesian diets, but was probably consumed during famine [7,8,9,10]. Traditionally, noni bark and roots were used as dye or clothing, while medicinal usage of all plant parts, including leaves and fruits, were mostly restricted to treat wounds, infections, menstrual cramps, bowel irregularities, diabetes, high blood pressure or as a purgative [9,11]. Based on published literature, selected traditional and modern uses of noni are listed in Table 1, some of which are extensively reviewed elsewhere [7,9,10,12].

**Table 1:** Traditional and modern uses of noni.

Noni Plant Parts	Traditional Usage	Modern Usage ^a^ [ref]
Leaves	Topical burns, headaches, fevers, neonatal inability to breath, bone fractures, menstrual cramps, gonorrhea *, back pain *, insect infestations, boils, rheumatic pain, ulcers, gout, internal bleeding, ringworm, neuralgia * [7,8,9,13,14].	To treat tuberculosis, helmintic infections, oxidative stress, open wounds, hyperlipidemia, and as an anti-allergen [15,16,17,18,19,20,21].
Fruit	Sores in mouth, peeling or cracking of toes, boils, pimples, blood impurities *, kava intoxication, insect infestations, tuberculosis, diabetes, blotchy skin *, heart trouble, stomach pain *, menstrual cramps *, heartburns, sore throat [9,13,14,22].	To treat bacterial and viral infections, cancer, pain, diabetes, nausea, gastric ulcers, liver disease, immune disorders, neuronal damage, cognition, helminthic infections, or to reduce oxidative stress, inflammation, inhibit oxidation of macromolecules (antioxidant capacity), improve memory impairment, cerebral blood flow, hyperglycemic and drug-induced hepatotoxicity. Noni was used as an antipsychotic, anxiolytic, sedative, and hypnotic therapy and also to enhance food color stability [16,21,23,24,25,26,27,28,29,30,31,32,33,34,35,36,37,38,39,40,41,42,43,44,45,46].
Bark	Stonefish poisoning, topical infections, asthma *, malaria [8,13,22].	No modern uses identified.
Root	Topical infections, stomach pain * [13].	To treat viral infections, hypertension, cancer, inflammation, hyperlipidemia, diabetes and pain [15,17,37,47,48].
Stem	Hernia [12].	Cure cutaneous leishmaniasis [49].
Seeds		To treat hyperlipidemia, oxidative stress or cancer [50,51,52].
Flower	Sore eyes, topical burns [8].	No modern uses identified.

^a^ Does not include anti-diabetic, clinical studies and anti-inflammatory, included in Table 2, Table 3 and Table 4 and Supplemental Table 2. NJ—Noni fruit juice; TNJ—Tahitian noni juice; * *Treated with combination of noni and other traditional medicines.*

Traditional healers prepared noni fruit juice (NJ) by pounding noni fruits to a pulp and straining juice through muslin cloth [9]. NJ was mixed with sugarcane juice and kukui nuts (*Aleurites moluccana* (L) Willd, Euphorbiaceae) to be used as purgative, or diluted with spring water to treat diabetes, high blood pressure or prevent intoxication from kava [9,13]. One of the most popular modern methods in Hawaiian households is to consume fermented noni fruit juice (fNJ), which is prepared by fermenting ripe or translucent noni fruits in airtight glass containers for more than two weeks in direct or indirect sunlight [9,10]. In contrast, commercial NJ (cNJ) may be extracted from raw or ripe fruits, with or without fermentation, and may be mixed with other fruit juices to reduce the bitter taste or mask the strong unpleasant odor of butyric acid present in ripe noni fruits [7,9,53]. Sometimes the fruits are chopped and dried after harvesting for ease of shipment to factories where the dried fruits are rehydrated to prepare juice [7]. Therefore, chemical constituents of commercial noni products may differ based on specific extraction procedures and ripeness of noni fruits. More than 200 noni phytochemicals and their bioactivities have been reviewed by Chan-Blanco *et al.* in 2006 [54], Pawlus and Kinghorn in 2007 [12], and Potterat *et al.* in 2007 [55]. Supplemental Table 1 summarizes bioactivities of chemicals isolated only from noni or its products that were identified after 2007. Additional commercial products include encapsulated freeze-dried NJ, concentrated extracts, powders, tinctures, and even fruit leather [9,10,53]. Types of commercial noni products, available for sale on the Internet, in local grocery stores, pharmacies or health food stores, are listed in supplemental Table 2. Terms used for the Internet searches were: “Commercial noni products” and “commercial types of noni juice sold for human consumption”. Only the first 100 sites were screened as examples of popular noni products, which are listed in supplemental Table 2.

In the 1990s, scientific claims of noni’s “healing powers” such as anti-inflammatory and anti-cancer properties fueled much of the commercial interest, promoting a worldwide market for noni-based dietary supplements including NJ [27,28,31]. Among the several health benefits of noni leaves, roots, seeds, fruits or their isolated chemicals [15,16,23,37,41,56,57,58,59,60,61,62], fNJ has been identified to improve glucose metabolism, increase insulin sensitivity, and prevent weight gain in diabetic animal models [23,34,63,64]. Although few clinical studies have investigated safety, anti-cancer, and anti-oxidant properties of Tahitian noni juice (TNJ) [65,66,67,68], laboratory findings of noni’s anti-diabetic potential have not translated into definite clinical outcomes. Overall, noni’s popularity is outweighed by scant clinical studies and marred by anecdotal incidences of hepatotoxicity [69,70,71,72,73,74]. The focus of this review is to summarize the anti-diabetic properties of noni and examine knowledge gaps in clinical outcomes.

**Table 2:** *In vitro* and *in vivo* studies in support of anti-diabetic properties of noni.

Noni Product (Source of Noni)	Relevant Study Outcome
*Anti-diabetic properties of fermented noni fruit juice (fNJ)*	
Noni juice prepared by fermenting fresh fruit pieces of *Morinda citrifolia* in water for three weeks (Trinidad and Tobago, West Indies)	fNJ significantly lowered blood glucose in streptozotocin-induced diabetic male rats and improved wound healing [64].
Noni juice prepared by fermenting fresh fruit pieces of *Morinda citrifolia* in water for six to 10 weeks (Trinidad and Tobago, West Indies)	fNJ significantly reduced fasting glucose and prevented liver degeneration in streptozotocin-induced diabetic male rats [23].
Fermented exudate from ripe noni fruits (fNJ, Honolulu, HI, USA)	fNJ inhibited weight gain and improved glucose and insulin tolerance and fasting glucose in high-fat diet (HFD)-fed C57BL/6 male mice in part by regulating hepatic forkhead transcription factor (FoxO1) mRNA expression [34].
*M. citrifolia* fruit powder (Bobsaewoo Seoul, Korea) fermented with Cheonggukjang containing soybeans, Bacillus sp. (KCTC 11351BP), *Bacillus subtilis* (KCTC11352BP), *Bacillus sonolensis* (KCTC11354BP) and Bacillus circulans (KCTC 11355BP)	➣ Fermented noni fruit powder was more effective in reducing fasting glucose, glycosylated hemoglobin (HbA1c), serum triglycerides and LDL cholesterol and improving insulin sensitivity in KK-Ay/TaJcl mice after 90 days as compared to diabetic controls and mice treated with non-fermented noni [63].
*Morinda citrifolia* fruit juice (Nigeria)	Lowered fasting blood glucose among alloxan-induced diabetic rats fed noni juice with or without insulin [75].
*Anti-diabetic properties of noni leaves*	
Methanol extracts of dried *Morinda lucida* leaves (Nigeria)	➣ Reduction of plasma glucose was significantly higher with *M. Lucida* extracts in streptozotocin-induced diabetic rats as compared to glibenclamide [76]. ➣ Significantly reduced plasma glucose levels in control Wistar male rats within four hours of administration. *Caution: May cause hypoglycemia*.
*Anti-diabetic properties of noni roots*	
Crude water, ethanol and butanol extracts of the dried roots of *Morinda officinalis* (Singapore)	➣ Crude ethanol extracts significantly reduced fasting glucose in streptozotocin-induced diabetic rats, but increased fasting glucose in normal rats. ➣ Water extracts demonstrated hypoglycemic effects in diabetic rats. ➣ Butanol fractions increased the fasting serum glucose levels in diabetic rats [77]. *Caution: May cause hypoglycemia or hyperglycemia*
Extracts butanol extracts of *M. citrifolia* roots, and two isolated anthroquinones viz., lucidin 3-*O*-β-d-primeveroside and morindone-6-*O*-β-d-primeveroside (Okinawa, Japan)	Significantly lowered blood glucose in streptozotocin-induced ddY diabetic male mice [78].
*Prevention of diabetes complications*	
*Morinda citrifolia* fresh fruit juice diluted in water (Okinawa, Japan)	Prevented neuronal diabetic complications such as infarction, neuronal damage, development of post-ischemic glucose intolerance and improved memory and insulin secretion in male ddY mice [60].
Ethyl acetate extract of shade dried *Morinda citrifolia* L*.* (noni) fruit powder (Bhuaneswar, India)	Noni fruit extract prevented streptozotocin-induced memory impairment in mice, due to reduced oxidative stress and acetylcholinesterase activity. Noni fruit extracts also increased levels of BDNF, acetylcholine, and ATP in brains of these mice [79].
Water, ethanol and chloroform extracts of *Morinda citrifolia* fresh fruits (Marathwada region, India)	Water extracts significantly inhibited rat lens aldose reductase (RLAR) activities and improved free radical scavenging activities *in vitro* as compared to ethanol and chloroform extracts thereby indicating a potential to inhibit cataract formation and prevent diabetic complications [80].
*Anti-diabetic mechanisms of noni*	
Methanol, n-hexane and chloroform extracts of *Morinda officinalis* roots and isolated compounds viz., alizarin-2-methyl ether, rubiadin-1-methyl ether and 1,2 dimethoxyanthraquinone (Chungbuk, Korea)	Extracts and isolated compounds increased adipocyte differentiation in 3T3-L1 mouse adipocytes as compared to insulin-treated cells demonstrating possible anti-diabetic effects [81].
Methanol extracts and four isolated compounds viz., episesamin 2,6-dicatechol, lirioresinol B, lirioresinol B dimethyl ether and ursolic acid from dried powder of *Morinda citrifolia*—plant part unidentified (commercially obtained from Babsaewoo Company in Suwon City, Korea)	Crude methanol extracts and isolated compounds were demonstrated to increase glucose uptake in 3T3-L1 mouse adipocytes, specifically by inhibiting protein tyrosine phosphatase 1B (PTP1B), known to induce insulin resistance when overexpressed [82].
*M. citrifolia* fruit powder (Bobsaewoo Seoul, Korea) fermented with Cheonggukjang containing soybeans, Bacillus sp. (KCTC 11351BP), *Bacillus subtilis* (KCTC11352BP), *Bacillus sonolensis* (KCTC11354BP) and Bacillus circulans (KCTC 11355BP)	Improved glucose uptake in cultured C2C12 mouse muscle cells, *in vitro* via stimulation of AMP-activated protein kinase (AMPK) [63].
Aqueous extracts of *Morinda lucida* leaf (Lagos, Nigeria)	Inhibited α-amylase *in vitro* that was isolated from α-*Aspergillus oryzae and* glucosidase from α-*Saccharomyces cerevisiae* [83].
Ethanolic extracts of *Morinda citrifolia* Linn. Leaves and fruits	Inhibited protein glycation *ex vivo* in plasma of diabetic patients [84].

### 1.1. Anti-Diabetic Properties of Noni

Among the 15 studies investigating the anti-diabetic effects of noni, six studies report anti-diabetic properties of NJ prepared from fruits, while the remainder of studies identified the anti-diabetic properties of extracts prepared from noni roots or leaves, reviewed in Table 2. Earlier studies indicated that NJ prepared by fermenting fresh fruit pieces of *Morinda citrifolia* in water for three weeks significantly lowered blood glucose levels in streptozotocin-induced diabetic male rats and improved wound healing [64]. An increase in hydroxyproline contents of the healing tissues, and presence of polyphenols, triterpenoids, tannins, carboxylic acids, and steroids, as well as *in vitro* antimicrobial activity of fNJ were speculated to contribute towards its anti-diabetic and wound healing properties [64]. Fermentation of noni fruits for longer periods (six to 10 weeks) also demonstrated similar hypoglycemic effects in streptozotocin-induced diabetic male rats and prevented liver degeneration [23]. In addition to blood glucose levels, fNJ was observed to improve body mass, liver glycogen contents, and liver degeneration and was comparable to the anti-diabetic medicine glibenclamide [23]. Studies from our laboratory indicated that fermented exudate collected from ripe noni fruits after two weeks was not only able to lower fasting glucose, but also inhibited weight gain and improved glucose and insulin tolerance in high-fat diet (HFD)-fed obese and diabetic male mice [34].

Similarly, noni fruit powder fermented with Cheonggukjang containing soybeans and bacteria such as *Bacillus* sp. (KCTC 11351BP), *Bacillus subtilis* (KCTC11352BP), *Bacillus sonolensis* (KCTC11354BP), and *Bacillus circulans* (KCTC 11355BP) was more effective in reducing fasting glucose, glycosylated hemoglobin (HbA1c), serum triglycerides and low-density lipoprotein (LDL) cholesterol, and improving insulin sensitivity in KK-Ay/TaJcl diabetic mice as compared to diabetic mice treated with non-fermented noni [63]. A recent study demonstrated that NJ synergistically augmented insulin action and improved fasting blood glucose among alloxan-induced diabetic rats, as compared with only NJ or insulin [75].

In addition to noni fruits, methanol extracts obtained from dried noni leaves significantly reduced plasma glucose in streptozotocin-induced diabetic rats more effectively than those treated with glibenclamide [76]. However, these methanol extracts also significantly lowered plasma glucose levels in control Wistar male rats within four hours of administration, indicating possible adverse hypoglycemic effects [76]. Similarly, crude water and ethanol extracts of dried roots of *Morinda officinalis* significantly reduced fasting glucose in streptozotocin-induced diabetic rats [77]. Interestingly, crude water extracts of dried roots increased fasting glucose in normal rats, while butanol fractions increased the fasting serum glucose levels in diabetic rats indicating possible adverse effects [77]. Crude butanol extracts of *M. citrifolia* roots and two isolated anthroquinones such as, lucidin 3-*O*-β-d-primeveroside and morindone-6-*O*-β-d-primeveroside, significantly lowered blood glucose in streptozotocin-induced ddY diabetic male mice [78].

Overall NJ, specifically fNJ, was more widely studied and effective in lowering fasting glucose, improving glucose tolerance and insulin sensitivity in diabetic animal models [23,34,63,64,75]. Contrary to NJ, extracts of noni leaves, roots or stems indicated possible adverse events in normoglycemic animals [76,77,78].

Besides anti-diabetic activity, noni leaf extracts and NJ also prevented diabetic complications in animal models such as improved would healing in diabetic rats [61,64]. NJ also prevented infarction, neuronal damage and development of post-ischemic glucose intolerance [60]. NJ from Japan improved memory in male ddY diabetic mice [60], while noni fruit extracts from India prevented streptozotocin-induced memory impairment in mice, possibly due to reduced oxidative stress and acetylcholinesterase activity [79]. Increased levels of brain-derived neurotropic factor (BDNF), acetylcholine, and ATP in the brains of mice were, in part, responsible for improved memory and brain function in diabetic mice [79]. Potential to prevent cataract formation was indicated by inhibition of rat lens aldose reductase (RLAR) activities and improved free radical scavenging activities *in vitro* by aqueous extracts of noni fruits [80]. Noni plant extracts inhibited the oxidation of LDL in human hepatoma cells, HepG2 [85], and prevented alcohol-induced fatty liver, hyperlipidemia, and hypercholesterolemia in mice and hamsters fed HFD and noni [86,87].

### 1.2. Anti-Diabetic Compounds in Noni

Studies have identified specific anti-diabetic compounds from noni roots, but not from noni fruits that have demonstrated the maximum anti-diabetic benefits as mentioned above. Two anthroquinones isolated from *M. citrifolia* roots *viz*., lucidin 3-*O*-β-d-primeveroside and morindone-6-*O*-β-d-primeveroside lowered blood glucose in streptozotocin-induced ddY diabetic male mice [78], while alizarin-2-methyl ether, rubiadin-1-methyl ether, and 1,2 dimethoxyanthraquinone isolated from *Morinda officinalis* roots increased adipocyte differentiation in 3T3-L1 mouse adipocytes as compared to insulin-treated cells, indicating potential anti-diabetic properties [81]. Similarly, episesamin 2,6-dicatechol, lirioresinol B, lirioresinol B dimethyl ether, and ursolic acid isolated from dried powder of *Morinda citrifolia* (plant parts unidentified) increased glucose uptake in 3T3-L1 adipocytes [82].

### 1.3. Anti-Diabetic Mechanisms of Noni

Several cellular and molecular mechanisms together contribute to lowering fasting and post-prandial glucose, as well as improving glucose tolerance and insulin sensitivity. Anti-diabetic drugs such as thiazolidinedione (TZDs), are known to stimulate adipocyte differentiation and enhance insulin sensitivity [88]. Although insulin sensitivity was not evaluated, methanol extracts of noni roots and three anthraquinones: 1,2-dimethoxyanthraquinone, alizarin-2-methyl ether, and rubiadin-1-methyl ether isolated from these extracts significantly increased differentiation of 3T3-L1 adipocytes *in vitro* [81]. Independent studies propose insulin mimetic activities of extracts derived from noni fruit, leaf, and commercial juice as indicated by increased glucose uptake in differentiated 3T3-L1 adipocytes, but had no additional or synergetic effect in insulin-stimulated cells [89]. Episesamin 2,6-dicatechol, lirioresinol B, lirioresinol B dimethyl ether and ursolic acid isolated from methanol extracts of dried noni plant dose-dependently increased glucose uptake in 3T3-L1 adipocytes which was comparable to rosiglitazone [82]. These noni-derived compounds also inhibited protein tyrosine phosphatase 1B (PTP1B), a known inducer of insulin resistance [82]. Overexpression of PTP1B results in whole body insulin resistance while PTP1B knockdown improves insulin sensitivity and inhibits weight gain in mice [90]. Similarly, ethanol extracts of fermented noni stimulated glucose uptake in C2C12-derived mouse myotubules [63]. The effect on glucose uptake in C2C12 myotubes was attributed to increased expression of AMP-activated protein kinase (AMPK), a known sensor of cellular energy [63]. Overall, the effect of noni or its chemicals on increased glucose uptake may contribute to improved peripheral insulin resistance, which are also noted *in vivo*, in HFD-fed obese mice and diabetic KK-Ay/TaJcl mice [34,63]. However, the effects of noni on PTP1B and AMPK *in vivo* remain unknown.

Another mechanism to improve postprandial glucose is to delay carbohydrate absorption from the intestine, which may help to regulate insulin release. This can be achieved through the inhibition of intestinal α-glucosidase and α-amylase enzymes. Line weaver-Burk plots indicated that aqueous extracts of *Morinda lucida* leaves competitively inhibited α-amylase activity and non-competitively inhibited α-glucosidase activity *in vitro* [83]. In addition to dietary absorption, an increase in plasma glucose is also associated with insulin’s inability to regulate hepatic gluconeogenesis. Insulin regulates hepatic glucose production via activation of Akt/phosphatidylinositol-3-kinase (PI3K) pathway and subsequent inhibition of forkhead boxO transcription factor 1 (FoxO1). Our studies have demonstrated that fNJ reduced hepatic gluconeogenesis in HFD-fed obese mice by inhibiting gluconeogenesis enzymes and specifically regulating FoxO1 mRNA expression [34]. Inhibition of hepatocyte fatty degeneration was also speculated as one of the mechanisms to lower plasma glucose among streptozotocin-induced diabetic rats [23]. In humans, ethanol extracts of noni leaves and fruits were capable of inhibiting protein glycation in plasma of diabetic patients, *ex vivo* [84]. Although chronic inflammation is considered a critical etiological factor in T2D, and several anti-inflammatory properties of noni have been demonstrated (summarized in Supplemental Table 3), studies investigating a direct correlation between anti-inflammatory effects of noni and amelioration of diabetes are lacking.

### 1.4. Human Studies in Support of Anti-Diabetic Potential of Noni

To date, human studies documenting anti-diabetic effects of noni consumption among T2D individuals are lacking. However, four population-based surveys have established the prevalence of noni use by traditional healers and diabetic individuals. In 2008, correlations between noni use and diabetes were first identified based on a dietary survey and ethnomedical questionnaire administered to residents of Kalo and Wanigela districts of Papua New Guinea [89]. Chewing betel quid was identified as an independent risk factor for diabetes in Papua New Guinea. Interestingly, prevalence of diabetes risk was reduced among betel quid chewers of Kalo district who simultaneously consumed guava bud (*Psidium guajava* L.) and noni, as compared to betel quid chewers of Wanigela residents who were ethnically identical, but did not consume guava bud and noni. An alternate proof of concept was provided by analyzing the effects of noni fruit, leaf extract and commercial NJ (Flora Manufacturing & Distributing Ltd., Burnaby, BC, Canada) on glucose uptake in 3T3-L1 mouse adipocytes. Their studies indicated that noni fruit and leaf extracts increased basal glucose uptake, but had no synergistic effect on insulin-stimulated glucose uptake in 3T3-L1 adipocytes [89].

An ethnobotanical survey conducted among traditional medical practitioners, herbalists, and herb vendors of Lagos State in Southwestern Nigeria indicated that the juice from noni leaves, administered twice daily for 12–16 weeks was among the principal traditional herbal therapies to treat diabetes [91]. Another ethnopharmacological survey documented the use NJ obtained from peeled and crushed fruits by the majority of diabetic population in Mauritius and was also prescribed/recommended by the majority of traditional healers [92]. Responders also claimed to use NJ to treat diabetic neuropathy, diabetic dyslipidemia, and hypertension. One adverse event was reported in the form of diarrhea with the concomitant use of noni and Atrovastatin to treat hypercholesterolemia [92]. However, the actual therapeutic outcomes, in both these surveys, were not specified. In contrast a recent ethnopharmacological survey from the Republic of Palau indicated that noni had no effect on lowering blood glucose among the two users, but was effective for weight loss among all 15 users and lowered high blood pressure among 42% of the users [93]. However, these observational studies and surveys had incomplete information regarding plant parts used, method of preparation or consumption, duration of treatment, and/or exact dose of noni used to treat diabetes (Table 3).

**Table 3:** Human studies in support of anti-diabetic properties of noni.

Type of Noni Products, Dosage and Duration	Study Rationale	Subject Demographics	Study Outcomes [ref]
Type of noni product, dosage and duration unknown.	Population based observational study in Papua New Guinea.	365 participants from three provinces of Papua New Guinea Above 16 years of age.	Habitual intake of noni was protective against betel quid-associated T2D [89].
Juice from *Morinda lucida* leaves mixed with juice from *Saccharum officinarum* leaves and water administered twice daily for 12–16 weeks. Exact dosage of noni juice unknown.	Ethnobotanical survey to identify medicinal plants used to treat diabetes.	100 participants from five central districts of Lagos State in southwestern Nigeria consisting of traditional medical practitioners, herbalists and herb sellers (76% responders were males).	*Morinda lucida* was among the principal anti-diabetic plants to be used in traditional therapy to treat diabetes. Anti-diabetic outcomes were not specified [91].
One cup of *Morinda citrifolia* juice obtained from peeled and crushed fruits, three times a week.	Document ethnopharmacological data regarding the use of natural resources among diabetic population of Mauritius.	320 diabetic patients (42% males and 58% females) 20 traditional medicine practitioners (55% males and 45% females).	*Morinda citrifolia* was documented to be most widely used by both, diabetic individuals and traditional medicine practitioners to treat diabetes. Perceived to be beneficial in treating diabetic neuropathy, diabetic dyslipidemia, and hypertension [92].
Among 58 users of *Morinda citrifolia* L., 26 used leaves, 12 used stems, 4 used bark, 4 used roots and only 2 individual used fruits. Preparations consisted of decoction, juices or both. Exact dosage unknown.	Ethnopharmacological survey to identify correlation between use of traditional Palauan medicines and non-communicable diseases such as diabetes	520 individuals undertook the survey among which only 188 responses were conclusive. 61% responders were females and 18% were traditional healers.	Noni had no effect on lowering blood glucose, but effectively reduced weight among all 15 users and lowered high blood pressure among 42% of the users [93].

### 1.5. Clinical Studies Evaluating Safety and Health Benefits of Noni

There is no dearth of Internet testimonials attesting the miracles and health benefits of noni products, specifically NJ. However, clinical studies assessing medicinal properties of noni are limited. Table 4 summarizes clinical studies conducted using commercial or laboratory prepared noni products. Initial claim of noni’s anti cancer properties, reported in 2004, were based upon interviews with cancer survivors from Hawaii, as well as their medical records and pathological analysis [94], reviewed in detail by Brown [10]. In brief, a 69-year-old man diagnosed with poorly-differentiated invasive adenocarcinoma of the colon, and assigned to hospice care, demonstrated improved bodyweight and appetite after consuming fNJ for six months. Follow-up endoscopy, after six years of continued fNJ consumption, did not reveal disease progression [94]. The second patient, another 64 year old male, diagnosed with adenocarcinoma at the esophagogastric junction, survived for 16 years without recurrence of the disease, which is rare. Long-term, cancer-free survival was attributed to consumption of homemade fNJ. In both these cases immunomodulatory effects of fNJ were hypothesized to prevent metastases, thereby promoting long-term survival [94].

**Table 4:** Clinical studies demonstrating safety and health benefits of noni.

Type of Noni Products, Dosage and Duration	Study Rationale [ref]	Subject Demographics and Outcomes
Home made NJ Method of preparation and dosage—not reported Consumption duration—6 months in case 1, unknown in case 2	Case report based on 2 cancer patients interviews, review of medical records and pathological slides [94].	Case 1Male 69 years old Poorly differentiated invasive adinocarcinoma Case 2Male 64 years old Adinocarcinoma at the esophagogastric junction Overall, study indicated better disease control and survival outcomes
Freeze dried whole noni fruit 500 mg capsules, incremental dosage up to 12 g/day (Innovative Nutriceuticals and Noni Maui). Minimum of 28 days at a specified dose level.	Phase I clinical trials to investigate safety and toxicity of high noni doses and determine anti-cancer effects [95].	Advanced stage cancer patients without any standard treatments. ClinicalTrials.gov identifier: NCT00033878 No toxicity was observed until 28 days. Improved quality of life at doses of 6–8 g/day. Noni consumption did not affect tumor regression, but one patient with advanced stomach cancer noted no disease progression for 40 months during noni consumption.
TNJ, Morinda Inc., Provo, UT, USA 30, 300 and 750 mL/day for 28 days	To investigate clinically safe dose of TNJ in humans [67,68,96].	28 males and 68 females 18 to 64 years old. No significant effect on plasma lipids, glucose as well as liver and kidney function tests.
NJ, Morinda^®^, Provo, UT, USA Two oz., morning and evening for three months	Improving quality of life, bone mineralization and auditory (hearing) function [97].	Three participants in placebo group and five in NJ group. Postmenopausal women suffering from osteoporosis and hearing loss. Limited improvement in bone mineralization and hearing were observed.
TNJ, Morinda Inc., Provo, UT, USA 1 and 4 oz./day for one month	Investigate effects of aromatic DNA adducts among smokers [66].	203 heavy smokers, 18 to 65 years old. TNJ reduced aromatic DNA adducts levels in heavy smokers.
TNJ, Morinda Inc., Provo, UT, USA 29.5 mL and 118 mL/day for 30 days	Investigate anti-oxidant properties of TNJ [65].	285 heavy smokers, 18 to 65 years old TNJ reduced plasma superoxide anion radicals and lipid hydroperoxide in smokers.
Tahitian Noni Original^®^ Bioactive™ along with probiotics daily for 12 weeks, 30 min of daily exercise, calorie and dietary restrictions. Exact amount of noni juice consumed is not specified.	Determine the effects of noni and exercise on body fat composition and weight loss [98].	42 overweight male and female volunteers 16–65 years old, with BMI above 25 kg/m^2^. All participants lost nine to 12% of body fat and noted an average reduction of 1.2 to 2.5 kg/m^2^ BMI. Overall males lost more weight than females.
TNJ, Morinda Inc., Provo, UT, USA. 29.5 mL once in the morning (*n* = 51) or 59 mL twice daily-morning and night (*n* = 55) Placebo, 29.5 mL fruit juice devoid of iridoid glycosides and contained blend of grape and blueberry juices and natural cheese flavor to mimic the flavor of TNJ (*n* = 26) Total duration, 30 days	Investigate the effects of TNJ on cigarette smoke-induced dyslipidemia and systemic inflammation [99].	132 adult smokers, 18 to 65 years old Smoked more than 20 cigarettes per day, for more than one year. No concurrent use of prescription drugs or supplements. TNJ significantly reduced serum cholesterol, triglycerides, high-sensitive C-reactive protein (hs-CRP), LDL and homocysteine, and increased high-density lipoprotein cholesterol (HDL) among smokers.
TNJ, Morinda Inc., Provo, UT, USA. Dosage and duration unspecified.	Identify pre-operative use of herbal medicine among Nigerian outpatients [91].	Pre-operational cross-sectional survey conducted among 60 outpatients (55% females, 45% males) undergoing local, regional or general anesthesia.40% used several herbs for well being, 13% specifically for diabetes and 47% used herbs for hypertension. The exact number of patients using TNJ and the specific outcomes remains unknown.
Noni capsules containing 400 mg of pure milled noni herb powder (Vitamin World in Ronkonkoma, New York, NY, USA).	Investigate effects of noni on pain and inflammation in women suffering with dysmenorrhoea [100].	100 women participants suffering with dysmenorrhea, 18 years or older. Improvement in bleeding and pain scores was not significantly different in the noni group as compared to the controls.
Dried noni fruit extract 150, 300 and 600 mg one hour before surgery	Prevention of postoperative nausea and vomiting [36].	100 surgery patients, 18 to 65 years old. 600 mg noni fruit extract prevented postoperative nausea during first 6 h as compared to placebo.
Open-label, 2-period crossover study, with two weeks washout period and using single-dose of ethanolic noni fruit extracts and 300 mg ranitidine (1 tablet of Ranids). Dose of noni for humans was calculated according to the prokinetic dose of scopoletin obtained from the prokinetic investigation in rats.	Determine gastrointestinal motility and to elucidate gastrokinetic mechanisms [101].	10 each of nonsmoking healthy male and female volunteers, 18–45 years old, with BMI ranging from 18 to 25 kg/m^2^ Increased gastric mobility in healthy volunteers, possibly due to the ability of scopoletin to stimulate 5-hydroxyl tryptophan 4 (5-HT4) receptors as demonstrated in rats.
Noni leaf juice and ethanol extracts (30:0.05 *w*/*w*) was used at a dose of 0.6 mg/cm^2^	Investigating anti-allergenic and photo-protective properties of noni leaf extracts [102].	45 healthy subjects with limited skin pigmentation for allergy studies. 25 healthy individuals with Fitzpatrick skin type 2 for erythema studies. Noni leaf extracts prevented sodium lauryl sulphate-induced skin allergies and reduced ultraviolet ray B-induced erythema in healthy individuals.
*Morinda citrifolia* L. leaves, dosage, duration and method of preparation unidentified.	To document the use of medicinal plants among Nicobarese tribe from 15 villages of Andaman and Nicobar Islands [103].	70 Traditional Knowledge Practitioners 39% were 51–60 years old 27% were 41–50 years old 19% were 61–70 years old Leaves of *Morinda citrifolia* L. were mostly used for pains, aches, diarrhea, hypertension, fever, skin injuries, dental caries, hernia, snakebite, infertility, bone fractures and breathing difficulties.
Petroleum jelly-base ointment containing 1% methanol extracts of noni stems, applied three times a day up to six weeks.	Determine topical application to cure cutaneous leishmaniasis [49].	Forty patients (30 male, 10 female) with cutaneous leishmaniasis sores. 80% of the individuals showed positive response to treatment, while 20% had no significant improvement in sores. No side effects were noted.

The first clinical study evaluating health benefits of noni fruit was initiated in 2002 and completed in 2007. The phase-I clinical trial investigated the toxicity and efficacy of freeze-dried ripe noni fruit extracts among cancer patients to regress advanced tumors [104] and has been reviewed earlier [10]. Patients were randomized into five groups receiving two, four, six, eight or ten grams of noni/day for 28 days. While no adverse events were noted, significant reduction in pain was observed at all noni doses along with a non-significant dose response for global health status [95]. In spite of improvement in quality of life measures, noni did not demonstrate any therapeutic effects on tumor regression except for one patient with advanced stomach cancer who noted no disease progression for 40 months while consuming noni [95].

Although several varieties of NJ are available in the market, clinical safety assessments have so far been conducted only for Tahitian noni juice (TNJ, Morinda^®^, Provo, UT, USA). The first clinical safety study initially reported by Davies and Mugglestone in 2003 [96] and later reviewed by West *et al.* in 2006 [67] and Brown in 2012 [10] was a double-blinded, placebo-controlled trial with 96 healthy individuals consuming up to 750 mL of TNJ for six weeks. Consumption of TNJ for four and six weeks had no adverse effects on liver and kidney function tests, hematological analysis, differential red and white blood cell count, heart rate, and blood pressure [67,96]. Comprehensive analysis of the above study was subsequently reported in 2009 by West *et al.* [68], which included data collected at baseline (week 0) and two weeks, in addition to weeks four and six. Overall, these reports indicated that healthy individuals could consume up to 750 mL of TNJ daily for six weeks without any measurable side effects [67,68,96]. Clinical outcomes of TNJ were initially tested for its effects on hearing, bone turnover, and perceived quality of life in postmenopausal women [97]. Participants consumed 4 oz of TNJ daily (two ounces in the morning and evening) for three months. TNJ marginally improved bone remodeling and hearing in postmenopausal women [97]. The small sample size (three in placebo group and five in NJ group) was probably a major drawback to reach concrete conclusions.

Since 2009, TNJ has been methodically evaluated for its anti-carcinogenic [66], anti-oxidant [65], and weigh loss effects [98], as well as its ability to reduce cigarette smoke-induced dyslipidemia and systemic inflammation [99]. Randomized trials were conducted testing anti-carcinogenic and anti-oxidant effects of TNJ involving more than 200 heavy smokers consuming one ounce and four ounces of TNJ for one month. The anti-carcinogenicity study required the participants to consume TNJ along with one cup of water in the morning on empty stomach and demonstrated that aromatic DNA adduct levels were significantly reduced by 40%–50% as compared to baseline adduct levels at week 0 [66]. The second placebo-controlled, double-blinded study measured plasma superoxide anion radicals (SAR) and lipid hydroperoxide (LOOH) levels, pre- and post-intervention, among a total of 285 heavy smokers. While inter-group differences were absent in all the pre-tests conducted at baseline, the mean post-test levels of SAR and LOOH were significantly lower in TNJ groups compared to placebo groups, without any measurable side effects [65].

Consumption of TNJ in combination with probiotics, exercise, and calorie restriction for 12 weeks reduced overall body weights by 17.55 ± 9.73 lbs., reduced percent body fat by three to 15.4% and reduced average BMI by 2.6 ± 1.32 kg/m^2^ among otherwise healthy, but overweight individuals [98]. Men were noted to lose more weight than women. Although the authors present a compelling discussion regarding limited benefits of exercise and diet alone for weight loss, one of the major drawbacks of this study was the lack of appropriate control group receiving only noni or no probiotics, exercise, and calorie restriction [98]. A recent study among 132 heavy smokers, who smoked more than 20 cigarettes per day for more than one year, was a randomized, placebo-control trial [99]. Consumption of 29.5 mL to 118 mL of TNJ either once or twice a day was effective in significantly lowering serum cholesterol, triglycerides, high-sensitive C-reactive protein (hs-CRP), LDL and homocysteine, and increased high-density lipoprotein cholesterol (HDL) as compared to placebo juice devoid of iridoid glycosides [99]. In the same study population (subset sample size unspecified), TNJ also reduced cigarette smoke-induced lipid hydroperoxides (LOOHs)- and malondialdehyde (MDA)-DNA adducts [105].

Interestingly, TNJ was also identified in a cross-sectional survey, as one of the herbal products to be commonly used among Nigerian outpatients undergoing local, regional or general anesthesia [91]. Forty percent of the responders reported the use of several herbs for well-being, 13% specifically used herbs for diabetes, and 47% used them for hypertension. However, the exact number of out patients using TNJ, the dosage or the duration, as well as the specific outcomes or efficacy for overall herb usage, was not clarified [91].

In addition to TNJ, commercial noni capsules containing 400 mg of pure noni powder (Vitamin World, Ronkonkoma, NY, USA) demonstrated no beneficial effects on menstrual pain or bleeding when compared to placebo, in a recently-conducted, prospective, randomized, double-blinded placebo-controlled trial among women with dysmenorrhea [100].

Efficacy of non-commercial noni fruit extracts was tested in a prospective, randomized, double-blinded, placebo-controlled trial to prevent postoperative nausea and vomiting (PONV) [36]. One hundred elective surgery patients qualifying for three out of the four predictors of PONV, such as female gender, history of PONV, non-smoking, and opioid use, were randomized into four groups receiving either placebo or dried noni fruit extract capsules (150 mg, 300 mg or 600 mg) one hour before surgery. Number of episodes and severity of nausea and vomiting were recorded for 24 h post surgery. The highest dose of 600 mg noni fruit extract was effective in reducing the nausea in the first 0–6 h, but had no effect on postoperative vomiting after 6 to 24 h post surgery [36]. In contrast, an open-label, two-period crossover, kinetic study using ethanolic noni fruit extracts and 300 mg ranitidine (one tablet of Ranids), indicated that noni significantly increased gastric mobility in healthy volunteers, possibly due to the ability of scopoletin to stimulate 5-hydroxyl tryptophan 4 (5-HT4) receptors [101]. This study further suggests the possible use of noni fruit as a digestive, appetite-stimulating agent and to reduce bloating and heartburn [101].

Clinical studies have also evaluated the health benefits of noni leaves and stems [49,102,103]. Allergenicity tests of noni leaf extracts were conducted among 49 healthy individuals using sodium lauryl sulphate (SLS) as an adjuvant during the induction phase of the repeat-insult patch test. Similarly, efficacy of noni leaf extracts to ameliorate ultraviolet B (UVB)-induced erythema was conducted among 25 healthy subjects with Fitzpatrick skin type 2, that generally burns easily but tans minimally within the first 30–45 min of sun exposure. Skins of volunteers exposed to noni leaf extracts with or without SLS did not demonstrate any adverse reactions. Moreover, UVB dose required to induce erythema was 3.5 times higher at skin sites treated with noni leaf extracts compared to untreated skin [102]. Anti-inflammatory properties of noni leaf extracts were attributed to its inhibitory effects on histamine H-1 receptor binding activity, possibly due to the presence of the polyphenol quercetin in these extracts [102]. Similarly, petroleum jelly-base ointment containing 1% methanol extracts of noni stems significantly improved cutaneous leishmaniasis [49].

Among the 150 medicinal plant species recorded, leaves of *Morinda citrifolia* L. were recently identified, as having the highest medicinal-use value [103]. Traditional Knowledge Practitioners (TKP’s) of Nicobarese tribes from 15 villages of Andaman and Nicobar Islands used noni to treat pains, aches, diarrhea, hypertension, fevers, skin injuries, dental caries, hernia, snake bite, infertility, bone fractures, and breathing difficulties [103].

### 1.6. Bioavailability and Pharmacokinetics of Noni

Noni phytochemicals have been classified as flavonoids, lignans, iridoids, coumarins, anthraquinones, polysaccharides, terpenoids, sterols, and fatty acids [12]. It is expected that diverse biological activities and health effects of various noni preparations is based upon the concentrations and synergistic effects of its bioactive components.

So far, only two studies have evaluated bioavailability of noni or its bioactive components. The first pharmacokinetic study determined the bioavailability of scopoletin, a bioactive compound in noni among healthy volunteers. Five men and four women participated in the study and consumed either 1500 mg, 2000 mg, or 2500 mg of freeze-dried noni fruit in the form of 500 mg capsules in one day [106]. Although the sample size was extremely small, scopoletin was rapidly absorbed in the blood of all individuals within 0.3 to 0.5 h and excreted slowly in urine up to 8 h. Results indicate noni and its bioactive compounds assessed in this study are readily bioavailable in healthy individuals [106].

Recent study evaluated pharmacokinetics of noni iridoid glycosides, monotropein, and deacetylasperulosidic acid after oral administration of *Morinda officinalis* root extracts in rats [107]. Based on plasma concentrations, study indicated that monotropein had a shorter half-life and was eliminated faster than deacetylasperulosidic acid [107].

Several other bioactive components, such as the flavonoid quercetin (3,30,40,5,7-pentahydroxyflavone) and its glycoside, rutin (quercetin-3-*O*-b-rutinoside), have been independently demonstrated to be bioavailable in humans [108]. However, studies evaluating the pharmacokinetics and/or bioavailability of polyphenols, anthraquinones or other bioactive compounds not directly derived from noni or its extracts are beyond the scope of this review.

## 2. Discussion

Among the 469 studies identified by “PubMed” using the search term “*Morinda citrifolia*”, about 13 cell culture and animal studies have provided substantial evidence in support of noni’s anti-diabetic potential, its associated mechanisms, and its potential to mitigate diabetic complications [23,34,60,63,64,75,76,77,78,79,86,87]. Human studies in support of anti-diabetic potential of noni are restricted to four population-based surveys from the Pacific Islands and Africa, demonstrating the use of noni preparations by traditional healers and the general population to treat diabetes and its complications [89,91,92,93]. However, these surveys do not document or specify the effects of noni on T2D outcomes. Nevertheless, clinical studies investigating health benefits of noni, other than diabetes, are slowly emerging. It is encouraging to note that systemic evaluations of long-term safety, determination of efficacious doses, and randomized, placebo-control trials are being conducted for standardized commercial preparations of NJ, such as the TNJ [67,68,96]. Yet, several other commercial noni products in the market lack rigorous scientific evaluations for potential health benefits.

Although commercial preparations try to maintain quality control and consistency of dosage during the entire period of the product’s shelf life, differences in varieties of noni plants, conditions during cultivation, such as temperature, soil condition, nutrients, use of pesticides, stage of harvest, along with differences in methods of preparation may contribute to the differences in chemical composition of bioactive compounds in noni products. Few studies have tested and compared the chemical differences in commercial noni products. Study by West BJ *et al.* [109] determined the mineral profiles of 177 brands of commercial NJ according to a modified Association of Official Analytical Chemists protocol. Nine minerals were consistently identified in all NJ samples. Potassium was the most predominant mineral in all NJ and the range was consistent with those approved by European Union for TNJ (30–50 mg/serving) but was lower than potassium concentrations found in banana and yogurt.

Another study by Potterat *et al.* [110] compared the chemical composition of noni fruits, eight different cNJ, including TNJ, and four types of noni capsules from different parts of the world. Significant chemical differences were noted not only between juices and capsules, but also within different batches of same types of NJ [110]. A study by Samoylenko *et al.* identified six to seven similar chemicals in NJ obtained from Puerto Rico and Japan. However, scopoletin was detected only in freshly-squeezed NJ from Japan but was absent in NJ obtained from Puerto Rico [111].

Several classes of secondary metabolites, including polysaccharides, fatty acid glycosides, iridoids, anthraquinones, and flavonoids are present in NJ, which are metabolized in the liver by enzymes, such as cytochrome P450 (CYP), and may share the same metabolic pathways with prescription drugs [112]. In rats, quercetin and rutin present in noni was observed to significantly inhibit P-glycoprotein (P-gp) and CYP3A4, which are pharmacologically important in drug transport and metabolism, respectively [113]. Similarly, NJ also inhibited p-nitrophenol glucuronidation in rats, indicating reduction of UDP-glucuronosyl transferase (UGT), an enzyme involved in drug detoxification [114]. Herbal preparations including noni products are considered safe by the public based on their “natural origins”. Hence, herbal toxicities are difficult to diagnose, as patients do not report the use of natural supplements to their healthcare providers. So far, 11 noni-associated toxicity cases have been reported between 2000 and 2015 and nine of these occurring up to 2011 have been reviewed in detail earlier [10,115]. A recent toxicity report in 2012 was associated with consumption of Euforia juice that contained NJ along with other natural products. A 45-year old woman, consuming Euforia for one month to treat systemic sclerosis, eventually developed jaundice and presented elevated serum transaminases [74]. She was eventually diagnosed with hepatocellular necrosis and histopathological changes indicated toxic hepatitis. Her condition improved 18 months after discontinuing Euforia juice. Exact toxicity mechanisms remain unspecified, but several known hepatotoxic natural products including noni were hypothesized to induce herb-herb interactions [74].

NJ consumption for one week was associated with severe herb-drug liver toxicities in a 38-year old women concomitantly consuming low doses of phenobarbital for seven months to prevent electroencephalogram-associated seizures [73]. Although the patient was identified as a fast metabolizer of CYP2C9 and CYP2C19, phenobarbital-associated hepatic toxicities were not observed for seven months. All other causes of liver injury, such as viral hepatitis (A, B, C), cytomegalovirus, Epstein-Barr virus, human immunodeficiency virus, herpes simplex virus, autoimmune hepatitis, hemochromatosis, and Wilson’s disease were negative. Eventual discontinuation of both phenobarbital and NJ, along with steroid treatment, helped to normalize her liver abnormalities, which were hypothesized to be associated with “synergistic idiosyncratic herb-drug interactions” [73]. In a recent case report, poor seizure control in a 49-year old epileptic male was attributed to concomitant use of noni juice and phenytoin, a commonly-used drug for seizure control [116]. Reduction of noni juice consumption and addition of another anti-seizure medication helped to regain seizure control [116]. Although individuals and healthcare-providers have to be aware of possible noni-drug interactions, such interactions may also occur by consuming otherwise considered healthy food such as grapefruit [117].

Among the few positive clinical studies, TNJ was demonstrated to be safe among healthy individuals while additional clinical studies demonstrated reduction of oxidative stress, DNA adducts and dyslipidemia among heavy smokers consuming TNJ [65,66,67,105] and weight loss in healthy overweight individuals [98]. Commercial TNJ is a blend of 89% NJ and 11% mixture of grape and blueberry juices [54] and it is, therefore, difficult to credit NJ alone for the observed health effects.

Approval of NJ as a “novel food supplement” by European Commission was based on the non-toxicity and non-allergenicity of TNJ in adult rodents and safety tests conducted among healthy individuals. Considering the fact that hundreds of noni products are available in the market, clinical safety evaluations of TNJ does not necessarily endorse all commercial noni products as safe. Similarly, safety evaluations of TNJ performed among healthy individuals may not be applicable to individuals with chronic diseases and/or compromised immune systems as well as vulnerable populations, such as children, adolescents, and pregnant or breast-feeding women.

## 3. Conclusions and Future Directions

Successful clinical outcomes for noni products will depend on established and validated correlations between putative bioactive markers, molecular targets and specific health benefits. Although cell culture and animal studies provide compelling evidence in support of the anti-diabetic properties of laboratory-prepared fNJ, addressing the following knowledge gaps will be critical to determine the anti-diabetic potential of noni in humans.

(1) Appropriately designed randomized, placebo-control, double-blinded clinical studies in humans to determine bioavailability, safety, and long-term effects of noni and its bioactive components.
(2) Determine herb-drug interactions of noni, specifically with anti-diabetic medications.

Given the fact that multiple components of natural products can act synergistically to impart a greater biological effect than any given single component, there is an urgent need to develop techniques to not only identify the complex chemical components of noni products, but also establish a chemical or metabolite fingerprint for a specific biological effect. Furthermore, it is critical to understand that various noni products have diverse chemical profiles and may, therefore, have an assortment of biological effects and health benefits.

## Appendix

**Table A1:** Chemicals in diverse noni fruit preparations, including commercial NJ.

Type of Noni Products and Country of Origin [ref]	Identified Chemicals and Bioactivities
Laboratory prepared NJ, Okinawa, Japan [56]	A new anthraquinone, 1,5,15-tri-*O*-methylmorindol, and two new saccharide fatty acid esters, 2-*O*-(β-d-glucopyranosyl)-1-*O*-hexanoyl-β-d-gluropyranose and 2-*O*-(β-d-glucopyranosyl)-1-*O*-octanoyl-β-d-gluropyranose, were isolated from a methanol extract of noni fruits along with 10 known compounds, namely, two anthraquinones, six saccharide fatty acid esters, an iridoid glycoside, and a flavanol glycoside.
	*Bioactivities*: Anti-inflammatory and anti-cancer
➣ Noni fruits from Tahiti [118]	Scopoletin, rutin and quecertin, methanol, butanol
	*Bioactivities*: γ amino butyric acid (GABA) agonist activity *in vitro*
➣ Raw noni fruits from French Polynesia (Tahiti, Moorea, and Motu Fareone), Tonga, Dominican, Republic, Okinawa, Thailand, and Hawaii ➣ Commercial noni fruit juice from Tahiti, Dominican Republic, Hawaii, and Costa Rica ➣ Noni fruit powder capsule from French Polynesia, Hainan, South China Sea, Hawaii, and Indonesia [110]	Iridoid glucosides scopoletin, rutin, fatty acid glucosides, anthraquinones, asperulosidic acid, deacetylasperulosidic acid and rutin Bioactivities: not determined.
➣ Chemical constituents from the stems of noni plants (Karachi, Pakistan) [119]	morindicone (9-hydroxy-2-methoxy-4-methyl-3,10-anthracenedione), morinthone (4-methoxy-3-heptadecylxanthone), 1-hydroxy-2-methylanthraquinone and 2-hydroxymethylanthraquinone
	*Bioactivities*: not determined
➣ Chemical constituents from noni fruits (Karachi, Pakistan) [120]	New chemical:, morinaphthalenone Three known compounds: scopoletin, 1, 3-dimethoxy-anthraquinone and 1, 2-dihydroxy-anthraquinone
	*Bioactivities*: not determined
➣ Noni fruits (Taiwan) [121]	New anthraquinones: 1,6-dihydroxy-5-methoxy-2-methoxymethylanthraquinone and 1,5,7-trihydroxy-6-methoxy-2-methoxymethylanthraquinone, and one new lignin: isoamericanoic acid A. Known compounds identified: scopoletin, luteolin, americanin A, americanin D, 3,3′-bisdemethylpinoresinol, *p*-cresol, *p*-hydroxybenzoic acid, *p*-hydroxybenzaldehyde, 4-hydroxy-3-methoxybenzaldehyde, 4-hydroxy-3-methoxycinnamaldehyde, and 2,5-dihydroxy-4-methoxybenzaldehyde
	*Bioactivities*: not determined
➣ Chloroform-soluble extract of freeze-dried noni root powder obtained from Nature’s Sunshine Products, Inc. (Spanish Fork, UT, USA) [122]	Two new benzophenones: morintrifolins A and B, were isolated together with 14 known anthraquinones
	*Bioactivities*: four isolated known anthraquinones: 1,2-dihydroxyanthraquinone, 1,3-dihydroxy-2-methylanthraquinone, 2-hydroxy-3-(hydroxymethyl)anthraquinone, and 1,3,6-trihydroxy-2-methylanthraquinone induced quinone reductase (QR)-activity in Hepa lclc7 murine hepatoma cells
➣ NDichloromethane extracts of noni roots (kg. Tanjung Keramat, Langkap, Perak, Malaysia) [123]	Nordamnacanthal
	*Bioactivities*: not determined
➣ Volatile oils were isolated from samples of frozen, dried and roasted leaves (Society Islands of French Polynesia) [124]	Palmitic acid and E-phytol
	*Bioactivities*: Components of aromatic volatile oils from noni leaves
➣ Aqueous extracts of *Morinda officinalis* roots (China) [125]	Polysaccharides
	*Bioactivities*: increased bone mineral density, bone mineral contents and reduced serum cytokines in rats
➣ Noni fruits (Karachi, Pakistan) [126]	New compounds: Morinaphthalene and morindafurone Known compounds: 1,8-dihydroxy-6-methoxy-3-methyl-9-anthrone and 2,4-dimethoxy-9-anthrone
	*Bioactivities*: not determined
➣ Methanol extracts of noni leaves (French Polynesia) [127]	Rutin, kaempferol-3-*O*-α-l-rhamnopyranosyl-(1→6)-β-d-glucopyranoside, quercetin, and kaempferol
	*Bioactivities*: not determined
➣ Noni leaf and noni fruit extracts (Agriculture Park, Univ. Putra Malaysia, Malaysia) [128]	Catechin, epicatechin and rutin
	*Bioactivities*: Extracts inhibited lipoprotein lipase activity *in vitro*
➣ Ethanol extracts of *Morinda officinalis How* root powder (China) [129]	2-Hydr-oxy-1-methoxy-anthraquinone monohydrate
	*Bioactivities*: not determined
➣ Ethanol extracts of *Morinda officinalis How* root powder (China) [130]	3-Hydr-oxy-1,2-dimethoxy-anthraquinone
	*Bioactivities*: not determined
➣ Ethanol extracts of *M. officinalis* root powder (Zhaoqing city, Guangdong Province, China) [131]	Polysaccharides: an inulin-type fructan MP-1, and acidic polysaccharides: MP-2, and MP-3 consisting of galacturonic acid, arabinose and galactose
	*Bioactivities*: anti-fatigue activity in male Sprague Dawley (SD) mice
➣ Ethanolic extract of the roots of *Morinda officinalis* (Shanghai Hua Yu Chinese Herbs Co. Ltd.,Shanghai, China) [132]	Physicion (1), rubiadin-1-methyl ether (2), 2-hydroxy-1-methoxy- anthraquinone (3), 1,2-dihydroxy-3-methylanthraquinone (4), 1,3,8-trihydroxy-2-methoxy-anthraquinone (5), 2-hydroxymethyl-3-hydroxyanthraquinone (6), 2-methoxyanthraquinone (7) and scopoletin (8)
	*Bioactivities*: compounds 2 and 3 increased osteoblast proliferation, compounds 4 and 5 increased osteoblast alkaline phosphatase activity. All of the isolated compounds inhibited osteoclast tartrate resistant acid phosphatase activity and bone resorption. Compounds 1 and 5 strongly inhibited osteoclastic bone resorption.
➣ Noni root extracts (Kobe, Japan) [133]	New anthraquinone glycosides: digiferruginol-1-methylether-11-*O*-beta-gentiobioside; digiferruginol-11-*O*-beta-primeveroside; damnacanthol-11-*O*-beta-primeveroside; 1-methoxy-2-primeverosyloxymethyl-anthraquinone-3-olate; 1-hydroxy-2-primeverosyloxymethyl-anthraquinone-3-olat; and 1-hydroxy-5,6-dimethoxy-2-methyl-7-primeverosyloxyanthraquinone
	*Bioactivities*: not determined
Ethanolic extract from noni seeds (French Polynesia) [134]	Ursolic acid, 3,3′-Bisdemethylpinoresinol, americanin A, and quercetin
	*Bioactivities*: anti-oxidant, radical scavenging and inhibition of tyrosinase and elastase enzymes *in vitro*
Laboratory prepared ethanol extracts from powdered noni roots, Kelantan, Malaysia [135]	A new anthraquinone: 2-ethoxy-1-hydroxyanthraquinone Known anthraquinones: 1-hydroxy-2-methylanthraquinone, damnacanthal, nordamnacanthal, 2-formyl-1-hydroxyanthraquinone and morindone-6-methyl-ether
	*Bioactivities*: 1-hydroxy-2-methylanthraquinone and damnacanthal exhibited larvicidal activities against the larvae of *Aedes aegypti*.
Methanol extracts of noni fruits (Kampung Seronok, Penang, Malaysia) [136]	7-Hy­droxy-6-meth­oxy-2*H*-chromen-2-one
	*Bioactivities*: not determined
Laboratory prepared ethanolic extracts of noni fruits cultivated in Okinawa, Japan [57]	New iridoid glycoside: 9-epi-6α-methoxy geniposidic New hemiterpene glycosides: 3-methylbut-3-enyl 2′-*O*-(β-d-glucopyranosyl)- β-d-glucopyranoside (nonioside K), 3-methylbut-3-enyl 6′-*O*-(β-d-xylopyranosyl)- β-d-glucopyranoside (nonioside L), 3-methylbut-3-enyl 6′-*O*-(β-d-xylofuranosyl)- β-d-glucopyranoside (nonioside M) New saccharide fatty acid esters: 6′-*O*-(β-d-glucopyranosyl)-1′-*O*-[(2xi)-2-methylbutanoyl]-β-d-glucopyranose (nonioside N) and 6′-*O*-(β-d-xylopyranosyl)-1′-*O*-[(2ξ)-2-methylbutanoyl]-β-d-glucopyranose (nonioside O)
	*Bioactivities*: new and known compounds exhibited melanogenesis inhibitory activities in the α-melanocyte-stimulating hormone (α-MSH)-stimulated B16 melanoma cell without any toxicity.
Aqueous extracts of *Morinda morindoides* leaves (Abidjan, Côte d’Ivoire) [137]	Bioactive chemicals unidentified
	*Bioactivities*: antimicrobial efficacy against *Vibrio cholerae* O:1 *in vitro*
Methanol extract of *Morinda morindoides* fruits (Democratic Republic of the Congo) [138]	6-*O*-acetyl congener
	*Bioactivities*: anti-malarial activities against *P. falciparum*, but no cytotoxicity against the host KB 3-, human, black, cervix carcinoma cells *in vitro*
Ethanol extracts from powdered *Morinda officinalis* roots, Fujian Province, China [139]	Inulin-type oligosaccharides
	*Bioactivities*: not determined
Crude alcohol extracts from *Morinda morindoides* leaves, Kinshasa [140]	Epoxygaertneroside and Gaertneroside
	*Bioactivities*: *ex-vivo* spasmogenic and spasmolytic activities in ileums isolated from guinea pigs
Commercial TNJ [43,141]	Catechin, epicatechin, quercetin, and rutin, scopoletin, 5,15-dimethyl-morindol and (2*E*,4*Z*,7*Z*) diactorienoic acid
	*Bioactivities*: not determined
Noni fruits, source unknown [142,143]	Iridoids (deacetylasperulosidic acid and asperulosidic acid)
	*Bioactivities*: not determined
Laboratory prepared NJ, Costa Rica [16]	Coumarin, flavonoids, phenolic compounds and iridoids
	*Bioactivities*: reduced carrageenan-induced paw oedema, inhibited cyclooxygenase COX-1 and COX-2 activities, production of nitric oxide (NO) and prostaglandins E(2) (PGE(2)) in activated J774 cells
Commercial NJ, West Indies [23]	Flavonoids, triterpenoids, triterpenes and saponins
	*Bioactivities*: anti-diabetic and hepatoprotective properties *in vivo*
An aqueous extract of NJ, Thailand [35]	Scopoletin
	*Bioactivities*: prevented acid reflux esophagitis, reduced gastric lesions and accelerated the healing of chronic gastric ulcers in rats
Leaf and root oils from *Morinda lucida*, West Africa [144]	50 compounds identified from leaf oils mostly containing alpha-terpinene and beta-bisabolene, while root oil contained 18 compounds, the major constituents being 3-fluoro-*p*-anidine and hexadecanoic acid
	*Bioactivities*: anti-oxidant activity *in vitro*
Laboratory prepared methanol extract from noni fruits, Kumming, China [145].	Two new phenylpropanoids: methyl 3-(2,4-dihydroxy-5-methoxyphenyl) propionate, butyl 3-(2,4-dihydroxy-5-methoxyphenyl)propionate and one unusual propanoate,5-hydroxyhexyl 2-hydroxypropanoate
	*Bioactivities*: not determined
Tahitian noni juice [141]	Scopoletin, 5,15-dimethyl-morindol and (2*E*,4*Z*,7*Z*)deactrienoic ecid
	*Bioactivities*: Improved fecundity as well as facilitate pregnancy and fetal health in mice
*n-*Butanol extracts from ethanol soluble fractions of *Morinda citrifolia* fresh fruit, Hainan Province, China [146]	(2*E*,4*E*,7*Z*)-deca-2,4,7-trienoate-2-*O*-β-d-glucopyranosyl-β-d-glucopyra- noside and amyl-1-*O*-β-d-apio-furanosyl-1,6-*O*-β-d-glucopyranoside
	*Bioactivities*: not determined
Methanol extracts, from *Morinda citrifolia* fruits, Okinawa [147]	New saccharide fatty acid esters: nonioside P, nonioside Q, nonioside R, nonioside S, nonioside T and butyl 2-hydroxysuccinate (4-butoxy-3-hydroxy-4-oxobutanoic acid) as well as 26 known compounds
	*Bioactivities*: cytotoxic activities in human cancer cell lines (HL-60 and AZ521), inhibition of Epstein-Barr virus early antigen (EBV-EA) activation in Raji cells
Methanol extracts from fruits and leaves of *Morinda citrifolia* L. [148]	Scopoletin
	*Bioactivities*: extracts demonstrated anti-angiogenic activity in the chick chorioallantoic membrane assay.
Methanol extracts from ripe fruits of *Morinda citrifolia*, Andaman and Nicobar Islands, India [38]	Polyphenols, anthocyanin, carotenoids, tannins and ascorbic acid
	*Bioactivities*: anti-oxidant activity *in vitro*
Laboratory prepared methanol extracts from noni roots, Korea [81]	Three anthroquinones: 1,2-dimethoxyanthraquinone, alizarin-2-methyl ether and rubiadin-1-methyl ether
	*Bioactivities*: promoted adipocyte differentiation in 3T3-L1 mouse adipocytes
Ethanol extracts from noni fruit puree with seeds MO, USA [149]	Anthroquinones: alizarin, lucidin, rubiadin
	*Bioactivities*: not determined
Acetoacetate extracts from the roots of *Morinda* *officinalis*, Southern China [150]	Monotropein (iridoids glycoside)
	*Bioactivities*: anti-inflammatory, anti-apoptosis and anti-catabolic activity in chondrocytes with proposed role for cartilage protection during osteoarthritis
Laboratory prepared methanol extracts from *Morinda longifolia* leaves, Vietnam [151]	longifolides A and B
	*Bioactivities*: not determined
Laboratory prepared ethanolic extracts from *Morinda umbellata*, Taipei [152]	1,6-dihydroxy-2-methoxymethylanthraquinone, 6-hydroxy-7-methoxy-2-methoxymethylanthraquinone, 3,6-dihydroxy-7-methoxy-2-methylanthraquinone, and 6-hydroxy-2-methoxymethylanthraquinone
	*Bioactivities*: cytotoxic against human hepatoma cells, HepG2
Laboratory prepared methanol extracts from unripe noni fruit, Thailand [141,153]	Scopoletin, rutin
	*Bioactivities*: antidopaminergic and antiadrenergic *in vitro*
Ethanolic extracts of freeze-dried noni fruits and leaves obtained from French Polynesia [21]	Ursolic acid, rutin and kaempferol-3-*O*-α-l-rhamnopyranosyl-(1→6)-β-d-glucopyranoside
	*Bioactivities*: anti-allergenic (dermatitis) effects in mice
*Morinda officinalis* samples obtained from Xinyi, Maoming, Yangchun, Shaoguan, Xing’an, Meizhou, Deqing (Guangdong Province), Linga, Zhuhai (Hainan Province) and Baise (Guangxi Province), China [154]	Seven inulin-type oligosaccharides
	*Bioactivities*: not determined
*Morinda citrifolia* L. powder and lemon juice (5%, *w*/*v*), Equador [155]	➣ Flavanol glycosides: quercetin 3-*O*-rutinoside-7-*O*-hexoside quercetin 3-*O*-rutinoside-7-*O*-pentoside, quercetin 3-*O*-rutinoside and kaempferol 3-*O*-rutinoside. ➣ Anthraquinones: Lucidin. ➣ Flavones Glycosides: apigenin 6,8-di-*C*-glucoside, diosmetin 6,8-di-*C*-glucoside, diosmetin 7-*O*-rutinoside. flavanones ➣ Glycosides: Eriodictyol 7-*O*-rutinoside, Hesperetin 7-*O*-rutinoside. ➣ Xanthones glycosides: mangiferin, mangiferin gallate, iso mangiferin gallate
	*Bioactivities*: Anti-oxidant activities *in vitro*
Methanol extracts from *Morinda citrifolia* L. leaves, India [156]	44-coumaric acid, 4-hydroxybenzoic acid, vanillic acid, 4-hydroxybenzaldehyde, vanillin and ferulic acid
	*Bioactivities*: not determined
Pure damnacanthal chemical [157,158]	Damnacanthal is a known anthraquinone in noni.
	*Bioactivities*: regulation of cellular signaling pathways, inhibition of mast cell activation, release of granule compounds *viz.*, beta-hexosaminidase and tryptase as well as release of chemokine and cytokines from stimulated mast cells *in vitro* and anti-cancer activities in human hepatoma cells, HepG2
Extracts of *Morinda elliptica* Ridl stem (Pattani province, Thailand) [159]	One new anthraquinone: and 18 known anthraquinones
	*Bioactivities*: All demonstrated weak inhibitory activity against a susceptible strain of Staphylococcus aureus and a methicillin-resistant *S. aureus.* In addition, damnacanthal was also inhibited Microsporum gypseum and lucidin inhibited Entamoeba histolytica and giardia intestinalis.
*Morinda officinalis* *F. C. How* roots (Guangzhou Zhixin Pharmaceuticals Co. Ltd., Guangzhou, China) [160]	Oligosaccharide: Bajijiasu
	*Bioactivities*: improved male fertility in mice
Extracts of *Morinda tinctoria* leaves (Visakhapatnam, India) [161]	New compounds: cynarin and oleuropein
	*Bioactivities*: antioxidant properties *in vitro*
Chloroform fraction of *Morinda lucida* leaves (Mampong, Ghana) [162]	New tetracyclic iridoid, molucidin
	*Bioactivities*: anti-trypanosomal activity normal and cancer human cells *in vitro*

**Table A2:** Commercial noni products.

Noni Juice and Drinks	Noni Capsules Tablets, Soft Gels and Other Products
6 Blend Juice (Goji, Noni, Acai, Pomegranate, Mangosteen, Camu), LifeTime	Apollo noni capsules
Agrolabs Naturally Noni Organic Dietary Supplement Juice	Apollo noni powder
Apollo noni juice	Apollo noni tablets
Apollo noni energy drink	Big Island noni capsules
Big Island noni juice	Doctor’s Best Noni Concentrate
Biorganic Life™ Noni Juice	Dynamic Health Noni capsules
Biorganic Life™ Triple Strength Noni Supreme Juice with Acai	Hawaiian herbal blessings-noni capsules
Dynamic Health Laboratories, Inc. *Morinda citrifolia* High Potency	Noni Maui noni capsules
Dynamic Health Laboratories, Inc. *Morinda citrifolia* noni juice	Noni Maui noni powder
Dynamic Health Men’s Choice Vitality Formula Noni Juice	Puritan’s Pride noni capsules
Dynamic Health Noni for Men Vitality Formula	Spring Valley Noni Dietary Supplement Softgels
Dynamic Health Papaya with Apple Cider Vinegar and Noni	
Dynamic Health Women’s Choice Formula Noni Juice	Other Products
Earth’s Bounty Organic Tahitian Noni	Hawaiian noni Honey
Earth’s Bounty Tahitian Organic Noni Juice	
Genesis Today Organic NONI	
Hawaiian herbal blessings-noni juice	
Hawaiian Ola Noni Energy Drink	
Hawaiian Virgin noni juice	
Nature’s noni juice	
NHT Global noni juice	
Noni and goji juice blends, HIRO Energy	
Noni Energy	
Noni Mangosteen Goji Acai Juice Blend LifeTime 32 oz Liquid	
Noni Maui noni juice	
NOW Foods Organic Noni Superfruit Juice	
Puna noni juice	
Purva Vita Noni juice	
Raw noni juice	
Sammi Noni and Ginseng Energy Drink	
Tahitian noni juice	
Virgin Noni Juice	
Vitacost Organic Certified Tropical Noni Juice	
Wai Lana Raw Aged Hawaiian Noni Juice Nutritional Supplement	

**Table A3:** Studies demonstrating anti-inflammatory effects of noni.

Noni Product (Source of Noni) [ref]	Relevant Study Outcomes
Aqueous extracts of noni fruits (Jamaica) [163]	Inhibited bradykinin-induced paw inflammation in mice and rats
Four saccharide fatty acid esters and one anthraquinone isolated from a methanol extract of powdered noni fruit (Okinawa) [56]	➣ Reduced TPA-induced inflammatory ear edema after 6 h in female ICR mice. ➣ Inhibited TPA-induced activation of Epstein-Barr Virus after 48 h in Raji human lymphoblastoid cells.
Methanolic extract of Noni fruit powder [164]	➣ Reduced nitric oxide synthesis through inducible nitric oxide synthase inhibition after 48 h in LPS-activated murine periodontal macrophages.
Phytochemicals isolated from noni fruits (Tahiti) [165]	➣ Inhibited lipooxygenase activities in human peripheral blood mononuclear cells and rabbit reticulocytes. ➣ Reduced cyclooxygenase 1 and 2 enzymatic activity.
Alcohol extract of noni puree from ripe fruits (French Polynesia) [58]	➣ Reduced inflammation in human monocytes 24 h after LPS-stimulation by inhibiting MMP-9 release to a similar degree as hydrocortisone. ➣ Reduced pain sensitivity in a hot plate test in male NMRI mice. ➣ Overall reduce arthritis pain and joint degeneration in mice.
➣ Chloroform extract of powdered noni roots (Taiwan) [47] ➣ Damnacanthal, isolated from crude noni root extract	➣ Chloroform phase and damnacanthal reduced formalin-induced pain behavior in male ddY mice after 30 min. ➣ Chloroform phase and damnacanthal inhibited histamine-induced paw edema similar to diphenhydramine after 3 h. ➣ Damnacanthal displaced histamine from binding to H1 receptor in HEK-293 cells after 1 h.
Aqueous extracts of noni fruits (Jamaica) [163]	Inhibited bradykinin-induced paw inflammation in mice and rats
Four saccharide fatty acid esters and one anthraquinone isolated from a methanol extract of powdered noni fruit (Okinawa) [56]	➣ Reduced TPA-induced inflammatory ear edema after 6 h in female ICR mice. ➣ Inhibited TPA-induced activation of Epstein-Barr Virus after 48 h in Raji human lymphoblastoid cells.
Methanolic extract of Noni fruit powder [164]	➣ Reduced nitric oxide synthesis through inducible nitric oxide synthase inhibition after 48 h in LPS-activated murine periodontal macrophages.
Phytochemicals isolated from noni fruits (Tahiti) [165]	➣ Inhibited lipooxygenase activities in human peripheral blood mononuclear cells and rabbit reticulocytes. ➣ Reduced cyclooxygenase 1 and 2 enzymatic activity.
Alcohol extract of noni puree from ripe fruits (French Polynesia) [58]	➣ Reduced inflammation in human monocytes 24 h after LPS-stimulation by inhibiting MMP-9 release to a similar degree as hydrocortisone. ➣ Reduced pain sensitivity in a hot plate test in male NMRI mice. ➣ Overall reduce arthritis pain and joint degeneration in mice.
➣ Chloroform extract of powdered noni roots (Taiwan) [47] ➣ Damnacanthal, isolated from crude noni root extract	➣ Chloroform phase and damnacanthal reduced formalin-induced pain behavior in male ddY mice after 30 min ➣ Chloroform phase and damnacanthal inhibited histamine-induced paw edema similar to diphenhydramine after 3 h. ➣ Damnacanthal displaced histamine from binding to H1 receptor in HEK-293 cells after 1 h.
➣ Ethanol extract from fresh unripe fruits of *Morinda citrifolia* (Thailand) [37] ➣ Damnacanthal, an anthraquinone isolated from fruits	➣ Fruit extract reduced carrageenen-induced paw and EPP-induced ear edema in male Wistar rats and Swiss albino mice. ➣ Damnacanthal inhibited NFκB induction via cyclooxygenase 2/inducible nitric oxide synthase pathway after 12 h. in LPS-stimulated macrophages.
➣ Aqueous extracts of unripe noni powder (Songkhla province, Thailand) [35] ➣ Scopoletin isolated from fruits	➣ Accelerated healing of chronic gastric ulcers in male Wistar rats. ➣ Prevented acid reflux esophagitis, and reduced ethanol-induced acute gastric lesion *in vivo.*
Noni juice prepared by enzymatic digestion of fruit puree (Costa Rica) [16]	➣ Inhibited COX 1 and 2 enzymatic activity *in vitro.* ➣ Reduced interferon γ, nitric oxide and prostaglandin E2 levels in LPS-activated J774 macrophages after 24 h ➣ Inhibited carrageenan-induced paw edema for 1–24 h, following gavage or IP administration of noni juice or indomethacin.
Scopoletin, rutin and quercetin isolated from TNJ (Tahiti, USA) [166]	➣ Scopoletin and quercetin Inhibited nitric oxide in LPS-stimulated RAW 264.7 mouse macrophages. ➣ Scopoletin and quercetin induced quinone reductase activity in Hepa 1c1c7 mouse hepatocytes.
Monotropein isolated from *M. officinalis* roots (Korea) [167]	➣ Reduced iNOS, PGE2, COX-2, TNF-α, IL-1β protein levels and DNA binding activity of NFκB in RAW 264.7 LPS-activated macrophages after 24 h. ➣ Inhibited inflammation in (DSS)-induced colitis in male ICR mice after 18 days.
➣ Ethanol extract of Noni seeds (French Polynesia) [168] ➣ 3,3′-bisdemethylpino resinol isolated from extract	➣ Ethanol extract and 3,3′-bisdemethylpino resinol inhibited MMP-1 secretion in UVA-irradiated normal human dermal fibroblasts after 48 h. ➣ 3,3′-bisdemethylpino resinol reduced p38 and c-Jun-terminal kinase (JNK) phosphorylation 30 min post UVA-irradiation.
Methanol extract of noni roots (unknown location) [169]	➣ Inhibited the iNOS, COX-2, TNF-α and NFκB in LPS-stimulated RAW 264.7 macrophages. ➣ Reduced carrageenan-induced edema in rats
Juice obtained from *M. citrifolia* fruits fermented for one year and pasteurized (Xuejia District, Tainan City, Taiwan) [87]	➣ Reduced liver and visceral fat, serum/liver lipids and enhanced fecal lipid/bile acid excretion in high-fat diet-fed hamsters. ➣ Mechanisms involved increased hepatic antioxidant capacities and lowered hepatic COX-2, TNF-α, and IL-1β expressions, serum ALT values in high-fat diet-fed hamsters.
NJ prepared by fermenting the fruits for one year (Taiwan) [86]	➣ Inhibited oxidation by increasing hepatic TEAC and GSH in alcohol-diet fed male C57BL/6 mice after 4 weeks. ➣ Reduced P38, ERK 1/2, NFκB P65, iNOS, COX-2, TNF-α and IL-1β protein levels and inhibited TLR2/4 mRNA expression.
Commercial TNJ [105]	Reduced c-reactive protein (CRP) and homocysteine levels in heavy smoking humans after 30 days.
➣ Ethyl acetate extract of dried noni fruit powder (Taiwan) [170]	➣ Inhibited *H. Pylori-*induced inflammation in AGS cells *by* reducing CagA, TNF-α, IL-8, iNOS and COX-2 protein levels after 6 h.
➣ Colostro noni, commercial noni product, containing bovine colostrum and freeze-dried *Morinda citrifolia* [171]	➣ Increased cell turn over and IL-8 mRNA gene expression in cultured intestinal cell line, Caco2. ➣ Hypothesized to promote tissue repair and prevent gastrointestinal inflammation.
➣ Ethanol and ethyl acetate extracts of fermented noni fruit extracts (Taiwan) [172]	➣ The fermented noni fruit extracts promoted growths of probiotic bacteria, *Lactilobacillus* and *Bifidobacterium* and down-regulated the intracellular oxidation and inflammation in human intestinal, Caco-2 cells.
